# Invasions but not extinctions change phylogenetic diversity of angiosperm assemblage on southeastern Pacific Oceanic islands

**DOI:** 10.1371/journal.pone.0182105

**Published:** 2017-08-01

**Authors:** Gastón O. Carvallo, Sergio A. Castro

**Affiliations:** 1 Instituto de Biología, Facultad de Ciencias, Pontificia Universidad Católica de Valparaíso, Valparaíso, Chile; 2 Laboratorio de Ecología y Biodiversidad, Facultad de Química y Biología, Universidad de Santiago, Santiago, Chile; 3 Centro para el Desarrollo de la Nanociencia y Nanotecnología (CEDENNA), Santiago, Chile; Oklahoma State University, UNITED STATES

## Abstract

We assessed changes in phylogenetic diversity of angiosperm flora on six oceanic islands located in the southeastern Pacific Ocean, by comparing flora from two periods: the pre-European colonization of islands and current times. We hypothesize that, in the time between these periods, extinction of local plant species and addition of exotic plants modified phylogenetic-α-diversity at different levels (deeper and terminal phylogeny) and increased phylo-β-diversity among islands. Based on floristic studies, we assembled a phylogenetic tree from occurrence data that includes 921 species, of which 165 and 756 were native or exotic in origin, respectively. Then, we studied change in the phylo-α-diversity and phylo-β-diversity (*1 –Phylosor*) by comparing pre-European and current times. Despite extinction of 18 native angiosperm species, an increase in species richness and phylo-α-diversity was observed for all islands studied, attributed to introduction of exotic plants (between 6 to 477 species per island). We did not observe significant variation of mean phylogenetic distance (MPD), a measure of the ‘deeper’ phylogenetic diversity of assemblages (e.g., orders, families), suggesting that neither extinctions nor introductions altered phylogenetic structure of the angiosperms of these islands. In regard to phylo-β-diversity, we detected temporal turnover (variation in phylogenetic composition) between periods to flora (0.38 ± 0.11). However, when analyses were performed only considering native plants, we did not observe significant temporal turnover between periods (0.07 ± 0.06). These results indicate that introduction of exotic angiosperms has contributed more notably than extinctions to the configuration of plant assemblages and phylogenetic diversity on the studied islands. Because phylogenetic diversity is closely related to functional diversity (species trait variations and roles performed by organisms), our results suggests that the introduction of exotic plants to these islands could have detrimental impacts for ecosystem functions and ecosystem services that islands provide (e.g. productivity).

## Introduction

Human-mediated global change has deeply impacted biodiversity composition and ecosystems functioning [1]. High extinction rates [2,3] and the introduction of exotic species [4,5] are recognized as important agents of biotic changes because these factors modify the structures of communities and ecological processes [2,6]. Species imported by humans to areas far from their native geographic ranges have impacted the taxonomic [7] and the phylogenetic structure of communities [8], thus, exotic species alter direct (e.g. [9]) and indirect interactions among species (e.g. [10]). Conversely, human-mediated reduction of the geographic ranges of species has resulted in risk of extinction [3], threatened ecosystem functioning and ultimately has impacted human wellbeing [6]. As a result of local extinctions and species introduction, various communities are becoming similar in taxonomic composition both in space and time [11], a phenomenon known as biotic homogenization [12,13]. Although the effects of biotic homogenization have been studied mainly in regard to taxonomic composition, other components of biodiversity such as phylogenetic and functional diversity are also impacted by the loss of distinctiveness among assemblages [8,12].

The patterns of evolutionary relationships among taxonomic entities within local communities are used as proxies to infer assembly mechanisms which account for species composition at different spatiotemporal scales [14]. Among plants, the phylogenetic properties of local assemblages are a key component to understanding ecological processes such as successional dynamics [15,16], productivity [17], invasion processes [18,19], and biotic interactions [20, 21, 22]. Because phylogenetic properties are deeply connected to ecological processes and ecosystem functioning [23], we can assume that phylogenetic similarity among assemblages translates into functional similarity. Therefore, an important issue is determining the contribution of extinctions and introductions as mechanisms which generate phylogenetic similarities among floras [8].

Because both extinctions and introductions of exotic species are non-randomly distributed within local assemblages [24], the net effects of these processes in phylogenetic structure fall along a continuum, between two extremes: they could favour phylogenetic clustering (e.g., the species are from some overrepresented clades) or phylogenetic overdispersion (e.g., the species tend to be evenly distributed in phylogeny) [25]. As extinctions and invasions simultaneously occur within a region, local assemblages could begin to show phylogenetic similarities. For example, extinctions contribute to phylogenetic overdispersion among comparable plant assemblages, because species losses occur in phylogenetically diverse groups [8]. On the other hand, when assemblages experience introduction of exotic species from a particular widespread invasive clade (e.g., Asteraceae, Poaceae families), they tend to show phylogenetic clustering and a high degree of phylogenetic similarity [26, 27].

Oceanic islands have a high degree of endemism and uniqueness of flora [28], on account of their isolation and high vulnerability to human impacts [29–32]. Many oceanic islands have only been inhabited since the European expansion to other continents (ca. 1500), which allows to empirically assess the effects of human-mediated disturbance on biodiversity components. This is in contrast to assemblages of continental flora, where the simultaneous contribution of different anthropogenic drivers could confound their roles on biodiversity structure. Some studies have evaluated the role of extinctions and invasions in determining the phylogenetic structure of mainland regions [8] and oceanic islands (see [33] for an analysis based on the native flora). In this sense, when human-induced extinctions have happened intensely on islands, significant changes are observed in the phylo-diversity when comparing pre-European and current time compositions. On the other hand, because exotic plant introductions exceed plant extinctions, as result of trade and deliberate introductions [34], and leading to taxonomic similarity [29], human-induced introductions could contribute to reducing the phylo-β-diversity among oceanic islands in spite of geographic distance. To evaluate these predictions, we conducted a study that assessed potential changes in phylogenetic diversity of angiosperm flora across six islands from the eastern portion of the Pacific Ocean. Specifically, our study aimed to answer the following questions: (i) to what extent has phylo-α-diversity of angiosperm island flora changed between pre-European colonization and current times? (ii) Is there evidence of a decrease in the phylo-β-diversity of the angiosperm flora among the studied islands?

## Materials and methods

### The islands and their angiosperm flora

**Fig 1:**
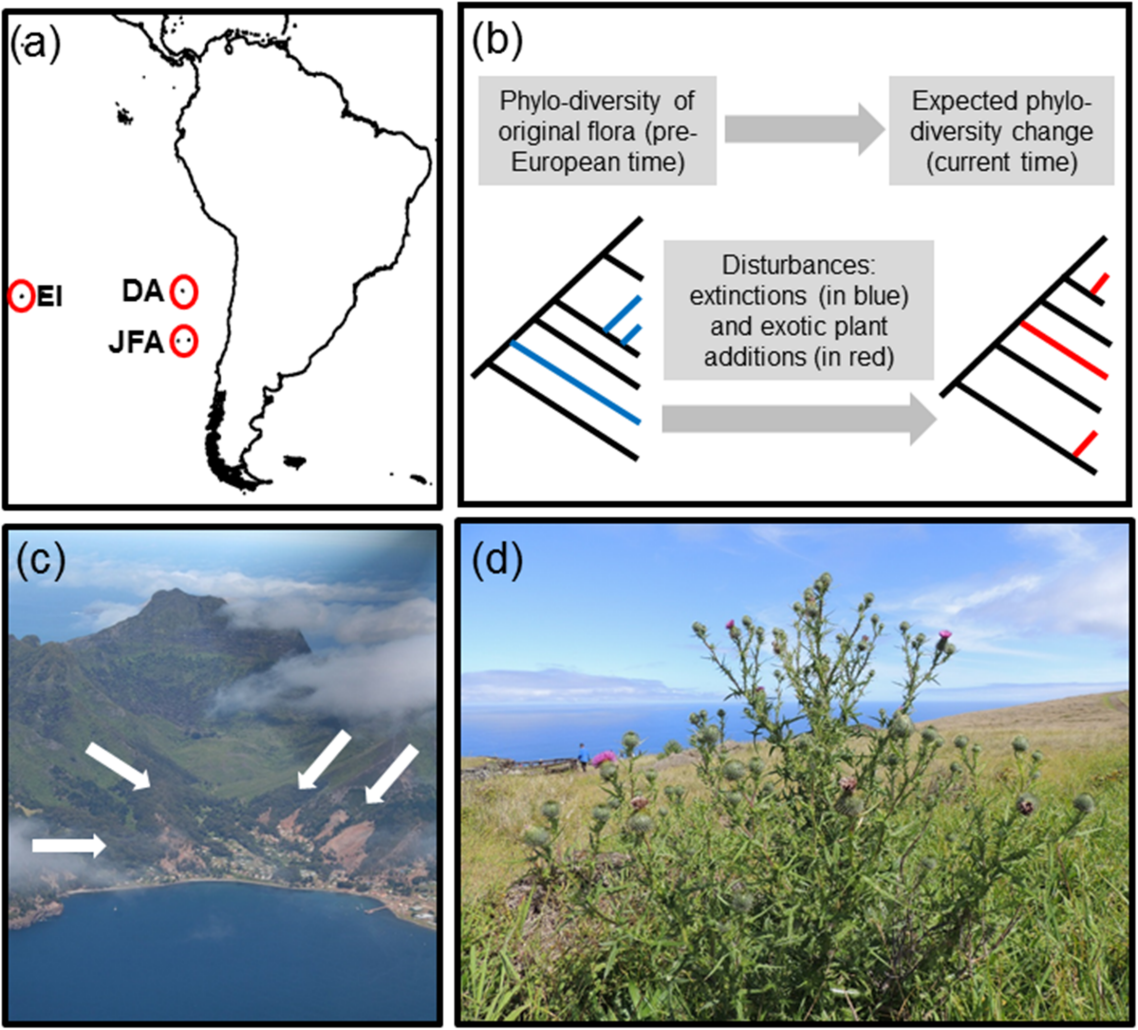
General aspects of the studied system. (a) A map shows the geographical location of the southeastern Pacific islands included in this study (red circles): Easter Island (EI), Desventuradas archipelago (DA) and Juan Fernández archipelago (JFA). (b) Graphical model summarizing potential effects of plant extinctions (blue branches) and exotic introductions (red branches) on the phylogenetic diversity of island flora. (c) An aerial view of Cumberland Bay at Robinson Crusoé Island, Juan Fernández archipelago; white arrows indicate presence of the dominant exotic trees *Eucalyptus globulus* (Myrtaceae) and *Ailanthus altissima* (Simaroubaceae); photograph kindly provided by G. Rojas. (d) *Cirsium vulgare* (Asteraceae) an exotic naturalized plant on Easter Island; photograph kindly provided by G. Rojas.

We examined the floristic composition of angiosperms of six oceanic islands located in the southeastern Pacific where taxonomic homogenization has been previously reported [35,36]. The studied islands are the only landmasses within 40 million km^2^ of water mass in this part of the Pacific Ocean [35]. These emerged from submarine volcanic plumes during the Plio-Pleistocene period, amounting to an area of approximately 350 km^2^ [37]. The studied islands (Fig 1) included the Juan Fernández archipelago (including Robinson Crusoé, Santa Clara, and Alexander Selkirk Island), Easter Island, and the Desventuradas archipelago (including San Ambrosio and San Félix Island). A more detailed description of the islands can be found in [35].

Using floristic studies, we assembled a database of the angiosperm flora on each island (S1 Table). Then, we checked that species in the database were present at the herbaria of the Museo Nacional de Historia Natural (SGO) and Universidad de Concepción (CONC). We did not detect new records in our database after reviewing herbaria sheets. After flora were compiled for each island, synonyms were removed and nomenclature was standardized by using the Plant List (www.theplantlist.org). Based on these recompilations, we divided angiosperm floras into two periods: (a) pre-European flora, which included the most likely composition of native angiosperm species on each island, and included extinct species, (b) current flora, which included actual composition of angiosperms with native and exotic plants, and excluded extinct taxa. For each island, compiled pre-European flora were exclusively composed of native species, including extinct angiosperms (locally extinct species without herbaria recorded within the last 50 years). The compiled current flora of each island included both native species (excluding those extinct) and exotic angiosperms. We considered exotic species as all naturalized and invasive plants according to criteria proposed by [38], based on independent reproduction for at least 10 years without human intervention [38]. Therefore, we considered as exotic angiosperm only those taxa absent in early floristic studies, and occurred at least twice in herbaria records with a span of 10 years between specimen registries.

Floristic studies considered to build our database depend on the individual study area. For the Desventuradas archipelago (DA), we have included information from the first botanical collection in 1869 [39] as well as that from subsequent expeditions [40–46] and taxonomical studies [47–49]. The Desventuradas archipelago has no permanent human population and, to date, no species have been reported as extinct on this archipelago. For Juan Fernández archipelago (JFA), we used information from the first collection that began in 1879 [50], complemented with the studies of Johow [51] and Skottsberg [52]. Although humans did not originally inhabit the islands of the JFA, since their discovery in 1574, they have held a permanent human population and, presently receive tourists. Among the Pacific islands, those of the JFA have one of the most comprehensive sets of floristic information available, which allows clear identification of exotic and native plants, as well as extinct taxa [48,53–60]. Regarding Easter Island (EI), inhabitation by Polynesian people modified flora, causing extinction of some plant taxa [61]. Botanical records are available since its discovery in 1722 and subsequent colonization [48,62–66], which allows distinction of native and exotic plants, and identification of extinctions following European colonization.

### Phylogenetic tree

Based on the checklist of species recorded for the islands, we assembled a phylogenetic tree with 921 angiosperm taxa using PHYLOMATIC [67] (http://www.phylodiversity.net/phylomatic/ accessed January 12, 2017) using tree version R20120829 is based on the APG III phylogenetic classification of angiosperms [68]. Within families, the phylogenetic relationships among genera were resolved through reordering by hand the obtained topology based on published phylogenies (the “graft” method, [69]) using Mesquite 2.74 (available at URL www.mesquiteproject.org). The full list of consulted references is provided in S2 Table. The resulting topology was age-calibrated based on the divergence times of angiosperms reported by [70] using the BLADJ algorithm implemented in PHYLOCOM [71]. We obtained a tree with 921 tips and 654 internal nodes, which accounts for 71% of the internal nodes resolved (S1 Fig). Complementarily, we assembled a tree exclusive to native plants (205 taxa) following the same methodology; in this tree, 74% of the internal nodes were resolved (S1 Fig). Although measures of community phylogenetic diversity are more sensitive to loss of resolution basally in the phylogeny, and less sensitive to loss of resolution terminally [72], our levels of unresolved nodes was relatively low and should not significantly impact estimated indexes. On the other hand, the use of PHYLOMATIC and the graft method is controversial (see [69] for an analytical criticism of this method).We chose this tool because several of our species are extinct or do not have genetic sequences available in GenBank.

### Estimation of phylo-α-diversity

Phylo-α-diversity was estimated for each island according to pre-European and current flora, based on a tree branch length index (Faith’s index [73]) and two distance-based indexes: the mean phylogenetic distance (MPD) and the mean nearest taxon distance (MNTD) [25]. Faith’s index is the sum of the branch lengths connecting all species in an assemblage [74]. The MPD is the mean of all phylogenetic distances that occur between all species pairs within an assemblage [25,75] and is interpreted as an indicator of the ‘deeper’ phylogenetic diversity of the studied flora [76,77]. The MNND is the mean of the phylogenetic distances that each terminal node has with their closest relatives within an assemblage [25,75,78]. It is interpreted as the ‘terminal’ phylogenetic diversity, which reflects the influence of relatively ‘recent’ events (e.g., local extinctions, migrations) on assemblage structure [77]. All these indexes were independently estimated for floras of the pre-European time (original flora, including extinct species) and current floras (actual flora composition, excluding extinct species and including exotic plants). In addition, indexes were estimated exclusively to native plant species for each studied period, to compare the contribution of extinctions on phylo-diversity. Indexes were estimated based on a calibrated tree (as a chronogram) and then expressed in millions of years. Because all island assemblages showed different levels of species richness (Table 1), indexes were standardized, which allowed removal of biases associated with differences in species richness [74]. Standardized effect size (SES) was estimated as *(observed–mean*_*null*_*)·(sd*_*null*_*)*^*-1*^, where *observed* is the directly estimated index, *mean*_*null*_ is the mean of the indexes obtained after 999 randomizations and *sd*_*null*_ is the standard deviation of the estimated indexes after randomization. Standardized MPD (MPD_SES_) and MNTD (MNTD_SES_) also as known as net relatedness index (NRI) and nearest taxon index (NTI), respectively [25, 78]. These indexes are estimators of phylogenetic clustering (NRI or NTI > 1) or overdispersion (NRI or NTI < 1) [25, 79]. We assessed whether 95% confidence intervals of NRI and NTI obtained from pooled island (N = 6 islands) were greater than 1.96 (phylogenetic clustering) or lower than -1.96 (phylogenetic overdispersion). For all standardizations, we randomized the phylogeny tip labels across all taxa included in the trees, we used this approximation to maintain the spatial structure of species in the system. Other constrained null models that focus on randomizing the community data matrix, rather than the phylogeny, generate that any spatial contagion or dispersal limitation is not maintained in the null community data matrices [74]. For all our estimations, we used the picante package [80] to R 3.0 (R Development Core Team).

**Table 1:** Angiosperms richness of six southeastern Pacific oceanic islands differentiating pre-European and current times.

		Pre-European	Current time		
Island	Location (lat; long)	Complete (all natives)	Complete (native plus exotic plants)	Native plants	Exotic plants
San Félix	26°17’ S; 80°05’ W	9	15 (67%)	9 (0%)	6
San Ambrosio	26°20’ S; 79°53’ W	19	25 (32%)	19 (0%)	6
Easter	27°06’ S; 109°20’ W	40	366 (815%)	26 (-35%)	340
Robinson Crusoé	33°38’ S; 78°50’ W	100	575 (475%)	98 (-2%)	477
Santa Clara	33° 42’ S; 78°56’ W	12	53 (342%)	12 (0%)	41
Alejandro Selkirk	33°45’ S; 80°47’ W	71	217 (206%)	69 (-3%)	148

Observed richness to current time differenced native and exotic species; values in parenthesis are percentage of change between compared periods. We also included the observed richness of exotic angiosperms (only current time).

We performed two kinds of analyses with the obtained indexes as well as plant richness. For the non-standardized indexes, we estimated the percent of change for each island by comparing the pre-European and current floras; we then assessed whether these percentages deviated from the null expectation (μ = 0) using a two-tailed t-test. For the standardized index (SES), we performed a paired t-test that contrasted the obtained values between the pre-European and current floras. These analyses were performed to compare pre-European and current times for complete floras and to only native species.

### Estimation of phylo-β-diversity

The phylogenetic-β-diversity (phylo-β-diversity, herein) was estimated as *1 –Phylosor*, where *Phylosor* is a similarity index that computes the fraction of shared phylogenetic branch lengths between two samples [81]. The phylo-β-diversity has values that range from 0 (phylogenetic homogenization between compared assemblages) to 1 (phylogenetic turnover between contrasted assemblages). The phylo-β-diversity was estimated (i) between times, by comparing each island between pre-European and current times, and (ii) among islands within each studied period. Phylogenetic differences among islands were correlated to geographical distance for each studied period, using a Mantel’s test [82]. In this way, we aimed to detect temporal or spatial phylogenetic turnover. All these analyses were performed on complete floras and on only native species. Because *Phylosor* is related to the species richness and the underlying taxonomic beta diversity [74], the observed *Phylosor* values were compared to null values generated by randomly shuffling taxa names in the phylogeny 999 times (*SES*_*Phylosor*_). During each iteration, the *Phylosor* was estimated for each island pair, and these null distributions were used to calculate the standardized effect sizes, where the mean of the null distribution was subtracted from the observed mean dissimilarity and then divided by the standard deviation of the null distribution [76]. Randomization procedure maintained the community presence–absence matrix. Because *SES*_*Phylosor*_ value is expressed in units of standard deviation, values between -1.96 to 1.96 indicate no-deviation from chance [74,83]. To assess the statistical significance of the changes (each island between times and among islands within each studied period), we contrasted the average of estimated phylo-β-diversity with a null expectation of μ = 0.

## Results

Overall, we observed a significant increase of 323% ± 120 in angiosperm richness (mean ± SE, N = 6 islands) when the complete pre-European and current floras were compared (Table 1) (result of t-test: t = 2.7, P = 0.021), with the greatest increase on Easter Island (815%) and the lowest on San Ambrosio Island (32%, Table 1). This increase was due to the addition of exotic angiosperms to islands (Table 1). The native flora showed, on average, a non-significant decrease in richness of 7% ± 6 (N = 6 islands; t = 1.7, P = 0.853); and three islands did not exhibit change in native angiosperm richness between the compared periods: San Félix, San Ambrosio and Santa Clara (Table 1). This result quantified local extinctions for islands, with Easter Island having the highest value (Table 1).

Among native plant species, we observed the greater occurrence for pre-European time as *Spergularia confertiflora* (Caryophyllaceae), which inhabited four islands: Alejandro Selkirk, Robinson Crusoé, Santa Clara and San Félix islands; in this period 81%, 17% and 2% of the 205 native plants occurred on only one, two and three islands, respectively (S1 Table). For current times, *Tetragonia tetragonoides* (Aizoaceae) showed the greater occurrence for five islands: Alejandro Selkirk, Easter, Robinson Crusoé, San Ambrosio and Santa Clara islands (S1 Table); in this period 78%, 18%, 3% and 0.5% of the 183 native species occurred on only one, two, three and four islands, respectively (S1 Table).

The tree-based phylo-α-diversity (Faith’s index) showed a significant increase of 157% ± 56 (N = 6 islands) to complete flora of angiosperms (t = 2.8, P = 0.039; Table 2), although we did not observe statistical changes for the native assemblage (-11% ± 9; t = 1.2, P = 0.276), in spite of local extinctions. Specifically, the highest changes of Faith’s index were for Easter, Robinson Crusoé, Santa Clara and Alejandro Selkirk islands (> 100% change, Table 2). Three islands (San Ambrosio, San Félix and Santa Clara) did not vary in Faith’s index to native flora, because their taxonomic composition did not change between compared times (Table 2). Concerning tree-based measure, the MPD showed non-significant differences between the pre-European and current floras to the complete angiosperm flora (-2.4% ± 1.4; t = 1.76, P = 0.139) as well as the native flora of angiosperms (-2.6% ± 2.6; t = 0.9, P = 0.373). The MNTD showed a significant decrease for angiosperm flora (-37% ± 6; t = 6.1, P = 0.002), although the native angiosperms did not show differences between the contrasted periods (-7% ± 7; t = 1.0, P = 0.359).

**Table 2:** Phylogenetic α-diversity (in millions of years) of angiosperm flora from six southeastern Pacific oceanic islands.

		PD			MPD			MNTD		
Islands	Period	Complete flora	Natives	Exotics	Complete flora	Natives	Exotics	Complete flora	Natives	Exotics
San Félix (DA)	Pre-European	889			227.9			156.7		
	Current	1,031	709	661	213.5	249.4	199.5	88.1	190.1	80.3
San Ambrosio (DA)	Pre-European	1,472			240.3			94.3		
	Current	1,607	1,151	1,003	225.5	240.9	210.5	76.8	135.5	108.1
Easter Island	Pre-European	2,849			261.2			111.9		
	Current	12,760	885	12,580	257.6	216.4	257.0	46.8	67.9	47.9
Robinson Crusoé (JFA)	Pre-European	4,701			259.4			57.7		
	Current	18,078	4,247	16,081	260.6	259.5	260.2	41.6	61.0	42.8
Santa Clara (JFA)	Pre-European	1,083			231.2			132.3		
	Current	2,802	935	5,432	236.0	231.9	238.3	69.3	131.6	73.6
Alejandro Selkirk (JFA)	Pre-European	3,725			265.4			60.6		
	Current	7,646	3,195	5,977	255.7	267.4	250.3	43.6	71.4	46.7

Phylogenetic diversity indexes reported for each period: Faith’s index (PD), mean phylogenetic distance (MPD) and mean nearest taxon distance (MNTD). Estimators to complete angiosperm flora and differentiating native and exotic plants (only current time). Abbreviations after island names depict archipelago name: Juan Fernández archipelago (JFA) and Desventuradas archipelago (DA).

**Fig 2:**
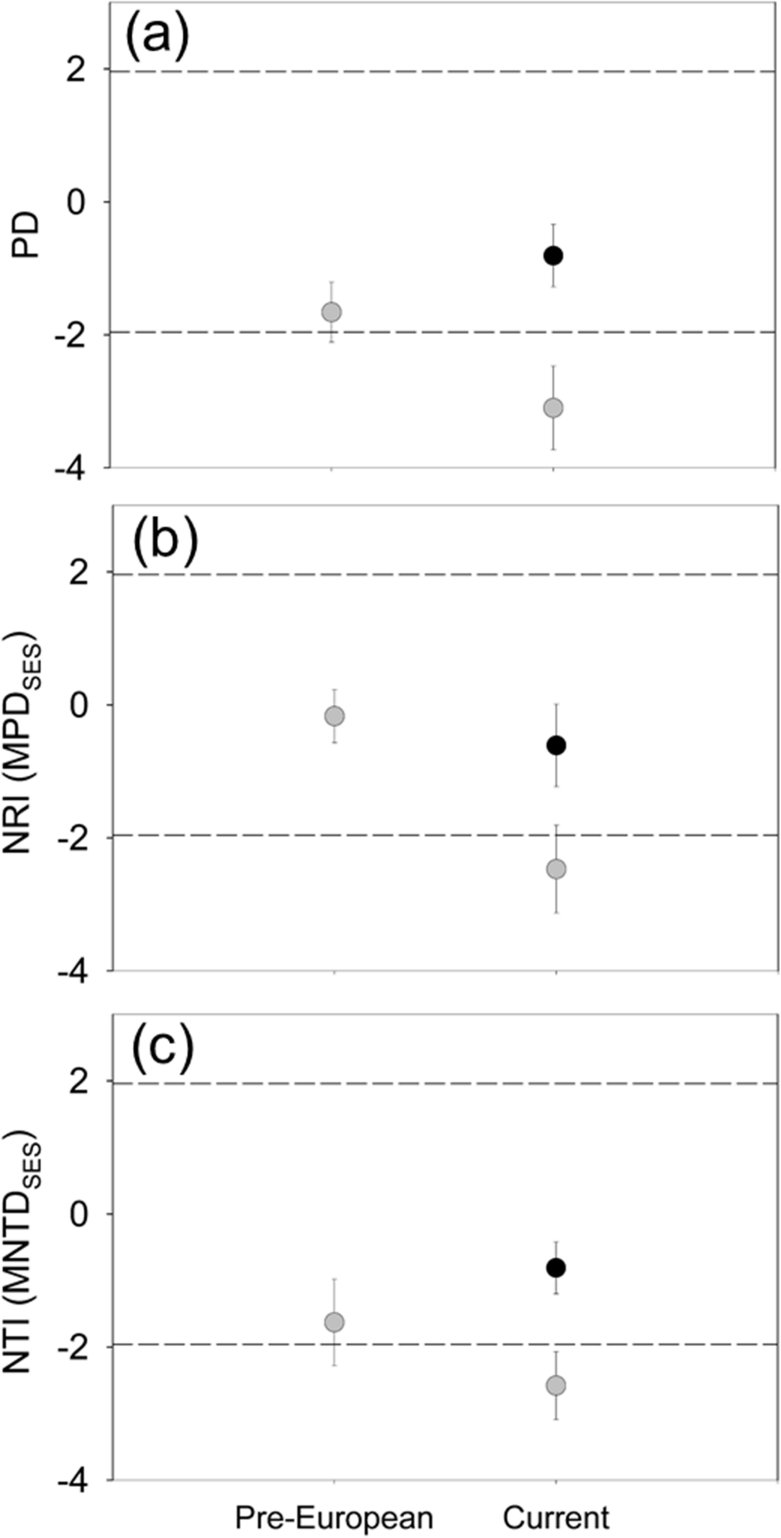
Standardized effect size (SES) of phylo-α-diversity indexes for pre-European and current time. (a) Faith’s index (PD), (b) net relatedness index (NRI, a MPD_SES_) and (c) nearest taxon index (NTI, a MNTD_SES_). Circles depicting mean of estimators (N = 6 islands) and deviations are ± 2 SE to complete flora (*grey* circles) and native flora (*black* circles). Dashed lines depict limits of null expectation; values above 1.96 suggest phylogenetic clustering, and below -1.96 indicate phylogenetic overdispersion.

Because phylogenetic diversity indexes are correlated to species richness, our analyses were also performed using standardized effect size (SES) indexes (Fig 2). For the standardized Faith’s index, we did not observe significant differences between the pre-European and current floras, neither for the complete flora (t = 1.9, P = 0.110, result of paired test) nor the native flora (t = 1.3, P = 0.252) (Fig 2A). The standardized MPD (net relatedness index, NRI) showed differences between the contrasted times for the complete flora (t = 3.9, P = 0.011) but not for the native flora (t = 0.9, P = 0.390) (Fig 2B). Lastly, the standardized MNTD (nearest taxon index, NTI) showed non-significant differences between the contrasted floras, both for the complete (t = 1.53, P = 0.187) and native flora (t = 1.19, P = 0.285) (Fig 2C), although values of complete flora indicates phylogenetic overdispersion for the current time (Fig 2C).

**Fig 3:**
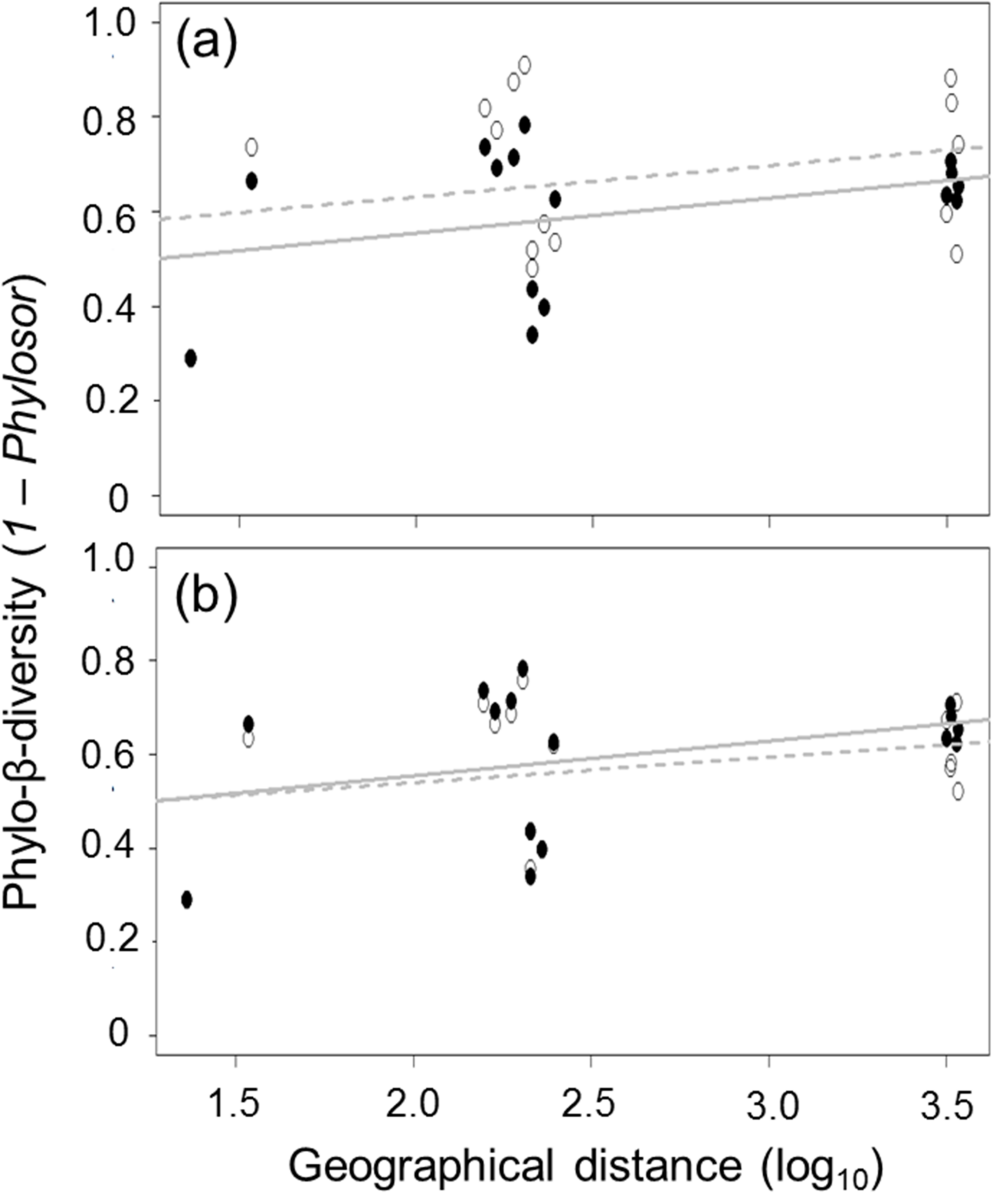
Relationship between phylo-β-diversity of angiosperms and geographical distance (log_10_). Complete flora (a) and only native plants (b) among southeastern Pacific Oceanic islands. For each panel, we show the relation and linear tendency splitting pre-European (*closed* circles and continuous line) and current time (*opened* circles and dashed line).

In regard to phylo-β-diversity, when complete flora of angiosperms were compared between pre-European and current times for each island, we observed a value of 0.38 ± 0.11 (mean of *1- Phylosor* ± SE; N = 6 pairs; result of contrast from null expectation μ = 0: t_1,5_ = 3.3, P = 0.010). However, the same comparison to native flora showed a difference of 0.07 ± 0.06 (N = 6 pairs) between compared times for each island; this difference did not deviated from null expectation μ = 0 (t_1,5_ = 1.3, P = 0.127). These results evidence an important change between the studied periods, for complete flora but not for native flora. In regard to change within each studied period, complete flora showed a phylo-β-diversity of 0.60 ± 0.04 to pre-European (N = 15 pairs; deviation from null expectation μ = 0 t_1,14_ = 15.0, P < 0.001) and 0.67 ± 0.05 to current time (N = 15 pairs; t_1,14_ = 14.1, P < 0.001). These results evidence spatial turnover among islands, although phylogenetic diversity was not coupled to geographic distance (Fig 3A) neither to pre-European (Mantel R = 0.292; P = 0.066) nor current time (Mantel R = 0.165; P = 0.073). To native flora within current time, phylo-β-diversity showed a value of 0.57 ± 0.04 (N = 15 pairs; deviation from null expectation μ = 0 t_1,14_ = 15.5, P < 0.001) and this did not exemplify a relationship to geographical distance among islands (Mantel R = 0.196; P = 0.218) (Fig 3B).

## Discussion

A recognized pattern in the face of the era of global change is that species introductions have contributed to increases in the taxonomic diversity of vascular plants [84], with the size of the flora of several islands increasing by as much as 100% [85–87]. This pattern, previously described for taxonomic diversity [35], is now also observed for the phylogenetic diversity, which increased by 157% between pre-European and current times (Faith’s index). Because native angiosperms did not significantly change between pre-European and current times (Faith’s index), our results suggest this change is mainly attributed to exotic plants. Increases in phylo-α-diversity did not occur at all levels, as we observed different results according to the index used. The index related to the ‘deeper’ structure of a phylogeny (MPD), which thus accounts for the phylogenetic diversity at the level of taxonomic orders or families, did not show statistically significant changes when the native flora of pre-European and current times were compared. This result indicates that any extinctions that occurred on these islands during the last ca. 500 years did not significantly erode phylogenetic diversity, and that invasions have contributed to homogenization of phylogenetic diversity of angiosperm assemblage. For example, Alejandro Selkirk and Robinson Crusoé Islands, which lost 7% and 3% of their native angiosperms between pre-European and current times, respectively, did not show any changes in MPD value. This could be attributed to extinctions that occurred within overrepresented clades, with phylogenetically related plant species remaining on the islands. In spite of the extinctions of *Podophorus bromoides* (Poaceae) and *Robinsonia macrocephala* (Asteraceae) on Robinson Crusoé Island, several native species of the Poaceae family remain (4 species) as well as species from the *Robinsonia* genus (6 species). Similarly, on Alejandro Selkirk Island, effects of the extinction of *Chenopodium nesodendrum* (Amaranthaceae) or *Empetrum rubrum* (Ericaceae) could be masked by other remaining species of the same families or orders on the islands, such as *Sarcocornia fruticosa* (Amaranthaceae) or *Gaultheria racemulosa* (Ericaceae). On the other hand, exotic species belonging to clades already present did not significantly contribute to changing the ‘deeper’ structure of the phylogenetic diversity on the studied islands. In light of these findings, the net effect of extinctions and introductions on the deeper phylogenetic structure could be considered neutral.

In terms of the ‘terminal’ phylogenetic diversity (MNTD), the diversity reflecting close relationships among specific taxa (e.g. species), we did not observe changes between the compared periods for the native flora (except on Easter Island); however, for the current time we observed a tendency to phylogenetic overdispersion of flora. On Easter Island, a long history of Polynesian occupation has left an impoverished landscape and a floristic composition that seem to be very far from the original [61,88]. This situation has caused extinctions that occurred within clades with few representatives, such as *Caesalpinia globulorum* and *Sophora toromiro*, both from Fabaceae and exclusive representatives of Fabales, as well extinction of *Elaeocarpus floridanus* (Elaeocarpaceae, exclusive representative of Oxalidales), *Macaranga* sp. (Euphorbiaceae) and *Xylosma suaveolens* (Salicaceae) representatives of Malpighiales. Also, the extinction of *Metrosideros collina* (Myrtaceae, the only member of Myrtales), *Paschalococcos disperta* (Arecaceae, the only representative of Arecales), *Potamogeton* sp. (Potamogetonaceae, from Alistamales), *Prenma serratifolia* (the only member of Lamiaceae and from the order Lamiales) and *Samolus repens* (Primulaceae the only representative of Ericales). The extinction of these plants importantly contributed to decreases in MPD and MNTD on Easter Island. However, the decrease in MNTD during current times for the other islands could be attributed to two non-exclusive potential explanations. First, the islands could have received species from similar widespread and diverse clades, such as Poaceae (84 exotic species from 49 genera), Asteraceae (72 species from 57 genera), Fabaceae (70 species from 40 genera), Malvaceae (22 species from 17 genera), Lamiaceae (22 species from 13 genera), Solanaceae (23 species from 9 genera) and Rosaceae (20 species from 12 genera), causing a decrease in the phylogenetic diversity and contributing to phylogenetic overdispersion. Secondly, environmental conditions that operate as both abiotic and biotic ‘habitat filters’ for exotics, such as climate, soil type or interspecific interactions [89], could contribute to maintaining phylogenetic overdispersion.

In regard to the phylo-β-diversity, we observed a greater differentiation among islands within periods, than between the studied periods for each island. Interestingly, the phylo-β-diversity of native species slightly decreased from pre-European to current times, which suggest that differences between times is mainly due to arrival of exotic angiosperms within recent decades. In addition, the increase of phylo-β-diversity for complete flora in the current time suggests that exotic species contribute to the phylogenetic homogenization of these islands.

We have described a significant change in the phylogenetic diversity among islands between two periods–one associated with the pre-industrial revolution, and one related to actual times. The post-industrial era has been associated with several global changes [3], among these, extinctions and invasions have shaped the biota on Earth. In light of our results, invasions have contributed more notoriously than extinctions to the configuration of plant assemblages. However, there are some caveats to our main findings, which should be considered in order to improve future studies on this topic. We used information regarding entire islands as assemblages, instead of evaluating sample units within each (e.g., plots), which could have excluded an important source of variation among the studied floras, since islands usually exhibit habitat heterogeneity, with variation in several biotic and abiotic conditions [31]. Additionally, geographical isolation and difficulty in accessing these islands are factors that contributed to our study being based on the use of databases, more than direct collections. In this context, our study focused on the presence or absence of plants, and conclusions may have differed if quantitative data had been used. Despite these limitations, our study showed a marked effect of exotic species on the phylogenetic diversity of the studied islands.

Insular floras represent vulnerable systems in terms of biodiversity conservation [29], and therefore, these are suitable for the evaluation of the effects of biotic homogenization. Insular areas exhibit a relatively small number of native species with an unbalanced representation of different taxonomic groups (i.e., phylogenetic over-dispersion) compared to continental areas [28,90]. These conditions suggest that, once subjected to biotic homogenization processes, insular areas must exhibit noticeable trajectories of change along their spectrum of phylogenetic information. As the introduction of new species adds new taxa non-represented in the communities, one consequence is phylogenetic overdispersion, mainly for terminal branches (species).

Recently, some authors have stressed the importance of reducing or preventing biotic homogenization at the biogeographic scale for conservation [91,92]. The phylogenetic structure and biogeographical relationships of insular floras constitute a feature of biodiversity, and therefore demand conservation efforts by their own merit [93,94]. Interestingly, evolutionary diversity may also reflect functional properties [8,14] and thus the capacity of species assemblages to respond to future environmental changes [78,95]. To date, conservation policies and efforts for the control of invasions have been independently developed on these islands. Although this approach can warn us about the extinction impacts of native species and notify the fate of future invasions, it is unlikely to stop floristic homogenization. This suggests the urgent need to coordinate conservation policies using a biogeographical approach to control phylogenetic homogenization as documented here.

## Supporting information

S1 Table Data matrix of studied flora, with angiosperm species names, status and occurrence on studied islands. (XLSX)

S2 Table List with all references used to resolve family polytomies. (DOCX)

S1 Fig Phylogenetic trees for angiosperm species registered in this study. (TIF)
