# Supplementary material for: Invasions but not extinctions change phylogenetic diversity of angiosperm assemblage on southeastern Pacific Oceanic islands
Source: PLoS One. 2017 Aug 1;12(8):e0182105. doi: 10.1371/journal.pone.0182105 (PMC5538740; doi:10.1371/journal.pone.0182105)
Supplement: S2 Table — (DOCX) [file pone.0182105.s002.docx]

**S2 Table.** A list with all references used to resolve phylogenetic polytomies

ACANTHACEAE

McDade LA, Moody ML (1999) Phylogenetic relationships among *Acanthaceae*: evidence from noncoding *trnL*-*trnF* chloroplast DNA sequences. *Am J Bot* 86:70–80.

Moylan EC, Bennett JR, Carine MA et al. (2004) Phylogenetic relationships among Strobilanthes *s.l.* (*Acanthaceae*): evidence from ITS nrDNA, *trnL-F* cpDNA, and morphology. *Am J Bot* 91:724–735.

ADOXACEAE

Winkworth RC, Bell CD, Donoghue MJ (2008) Mitochondrial sequence data and Dipsacales phylogeny: mixed models, partitioned Bayesian analyses, and model selection. *Mol Phyl Evol* 46:830–843.

AGAVACEAE

Good-Avila SV, Souza V, Gaut BS, Eguiarte LE (2006) Timing and rate of speciation in Agave (*Agavaceae*). *P Natl Acd Sci USA* 103:9124-9129

AIZOACEAE

Klak C, Khunou A, Reeves G, Hedderson T (2003) A phylogenetic hypothesis for the *Aizoaceae* (Caryophyllales) based on four plastid DNA regions. *Am J Bot* 90:1433–1445

AMARANTHACEAE

Kadereit G, Borsch T, Weising K, Freitag H (2003) Phylogeny of *Amaranthaceae* and *Chenopodiaceae* and the evolution of C_4_ photosynthesis*. Int J Plant Sci* 164:959–986.

AMARYLLIDACEAE

Meerow AW, Fay MF, Guy CL et al. (1999) Systematics of *Amaryllidaceae* based on cladistic analysis of plastid *rbcL* and *trnL-F* sequence data. *Am J Bot* 86: 1325–1345.

APIACEAE

Downie SR, Katz-Downie DS, Watson MF (2000) A phylogeny of the flowering plant family *Apiaceae* based on chloroplast DNA *rpl16* and *rpoc1* intron sequences: towards a suprageneric classification of subfamily Apioideae. *Am J Bot* 87: 273–292.

Downie SR, Spalik K, Katz-Downie DS, Reduron J-P (2010) Major clades within *Apiaceae* subfamily Apioideae as inferred by phylogenetic analysis of nrDNA ITS sequences. *Plant Div Evol* 128:111–136.

APOCYNACEAE

Alvarado-Cárdenas LO, Ochoterena H (2007) A phylogenetic analysis of the Cascabela-Thevetia species complex (Plumerieae, *Apocynaceae*) based on morphology. *Ann Missouri Bot Gard* 94:298–323.

Sennblad B, Bremer B (2002) Classification of *Apocynaceae* *s.l.* according to a new approach combining Linnaean and phylogenetic taxonomy. *Syst Biol* 51:389–409.

ARACEAE

Cusimano N, Bogner J, Mayo SJ et al. (2011) Relationships within *Araceae*: comparison of morphological patterns with molecular phylogenies. *Am J Bot* 98: 654–668.

ARALIACEAE

Wen J, Plunkett GM, Mitchell AD, Wagstaff SJ (2001) The evolution of *Araliaceae*: a phylogenetic analysis based on ITS sequences of nuclear ribosomal DNA. *Syst Bot* 26:144–167.

ASPARAGACEAE

Bogler DJ, Simpson BB (1996) Phylogeny of *Agavaceae* based on ITS rDNA sequence variation. *Am J Bot* 83:1225–1235.

Givnish TJ, Pires JC, Graham SW et al. (2006) Phylogenetic relationships of monocots based on the highly informative plastid gene *ndhF*: evidence for widespread concerted convergence. *Aliso* 22: 28 – 51.

Kim J-H, Kim D-K, Forest F et al. (2010) Molecular phylogenetics of *Ruscaceae* *sensu lato* and related families (Asparagales) based on plastid and nuclear DNA sequences. *Ann Bot* 106:775–790.

ASTERACEAE

Freire SE, Chemisquy MA, Anderberg AA, et al. (2014). The Lucilia group (*Asteraceae*, Gnaphalieae): phylogenetic and taxonomic considerations based on molecular and morphological evidence. *Plant Syst Biol* 301:1227-1248.

Funk VA, Anderberg AA, Baldwin BG, et al. (2009) Compositae metatrees: the next generation. In Funk VA, Susanna A, Stuessy TF, Bayer RJ, *Systemnatics, Evolution and Biogeography of Compositae*, International Association for Plant Taxonomy, Vienna, Austria, 747-777.

Liu P-L, Wan Q, Guo Y-P, Yang J, Rao G-Y (2012) Phylogeny of the genus *Chrysanthemum* L.: Evidence from single-copy nuclear gene and chloroplast DNA sequences. *PLoS One* 7: e48970.

Sancho G, de Lange PJ, Donato M et al. (2015) Late Cenozoic diversification of the austral genus *Lagenophora* (Astereae, *Asteraceae*). *Bot J Linn Soc* 177:78–95.

Schilling EE, Panero JL (1996) Phylogenetic reticulation in subtribe Helianthinae. *Am J Bot* 83:939 – 948.

Urbatsch LE, Baldwin BG, Donoghue MJ (2000) Phylogeny of the coneflowers and relatives (Heliantheae: *Asteraceae*) based on nuclear rDNA internal transcribed spacer (ITS) sequences and chloroplast DNA restriction site data. *Syst Bot* 25: 539–565.

BRASSICACEAE

Bailey CD, Koch MA, Mayer M et al. (2006) Toward a global phylogeny of the *Brassicaceae*. *Mol Biol Evol* 23:2142–2160.

BROMELIACEAE

Givnish TJ, Barfuss MHJ, Van Ee B, et al. (2011) Phylogeny, adaptive radiation, and historical biogeography in *Bromeliaceae*: insights from an eight-locus plastid phylogeny. *Am J Bot* 98:872–895.

CAMPANULACEAE

Eddie WMM, Shulkina T, Gaskin J et al. (2003) Phylogeny of *Campanulaceae* *s. str*. inferred from ITS sequences of nuclear ribosomal DNA. *Ann Missouri Bot Gard* 90:554–575.

CANNABACEAE

Yang M-Q, van Velzen R, Bakker FT et al. (2013) Molecular phylogenetics and character evolution of *Cannabaceae*. *Taxon* 62:473–485.

CARYOPHYLLACEAE

Fior S, Karis PO, Casazza G et al. (2006). Molecular phylogeneny of the *Caryophyllaceae* (Caryophyllales) inferred from chloroplast *matK* and nuclear rDNA ITS sequences. *Am J Bot* 93: 399–411.

CONVOLVULACEAE

Stefanovic S, Krueger L, Olmstead RG (2002) Monophyly of the *Convolvulaceae* and circumscription of their major lineages based on DNA sequences of multiple chloroplast loci. *Am J Bot* 89:1510–1522.

ERICACEAE

Kron KA, Judd WS, Stevens PF et al. (2002) Phylogenetic classification of *Ericaceae*: molecular and morphological evidence. *Bot Rev* 68:335–423.

EUPHORBIACEAE

Tokuoka T (2007) Molecular phylogenetic analysis of *Euphorbiaceae sensu stricto* based on plastid and nuclear DNA sequences and ovule and seed character evolution. *J Plant Res* 120:511–522.

Wurdack KJ, Hoffmann P, Chase MW (2005) Molecular phylogenetic analysis of uniovulate *Euphorbiaceae* (*Euphorbiaceae sensu stricto*) using plastid *rbcL* and *trnL-F* DNA sequences. *Am J Bot* 92:1397–1420.

FABACEAE

Brown GK, Murphy DJ, Ladiges PY (2011) Relationships of the Australo-Malesian genus *Paserianthes* (*Mimosoideae*: *Leguminoseae*) identifies the sister group of *Acacia sensu stricto* and two biogeographical tracks. *Cladistics* 27:380–390.

Sulaiman SF, Culham A, Harborne JB (2003) Molecular phylogeny of *Fabaceae* based on *rbcL* sequence data: with special emphasis on the tribe *Mimoseae* (Mimosoideae). *Asian Pac J Mol Biol Biotechnol* 11:9–35.

Wojciechowski MF (2003) Reconstructing the phylogeny of legumes (*Leguminosae*): an early 21^st^ century perspective. In Klitgaard BB, Bruneau A (eds), *Advances in Legume Systematics*, Royal Botanic Gardens, Kew, UK, 5–35.

IRIDACEAE

Souza-Chies T, Bittar G, Nadot S et al. (1997) Phylogenetic analysis of *Iridaceae* with parsimony and distance methods using the plastid gene *rps4*. *Plant Syst Evol* 204:109–123.

LAURACEAE

Chanderbali AS, van der Werff H, Renner SS (2001) Phylogeny and historical biogeography of *Lauraceae*: evidence from the chloroplast and nuclear genomes. *Ann Missouri Bot Gard* 88:104–134.

LYTHRACEAE

Chanderbali AS, van der Werff H, Renner SS (2001) Phylogeny and historical biogeography of *Lauraceae*: evidence from the chloroplast and nuclear genomes. *Ann Missouri Bot Gard* 88:104–134.

MALVACEAE

Alverson WS, Whitlock BA, Nyffeler R et al. (1999) Phylogeny of the core Malvales: evidence from *ndhF* sequence data. *Am. J Bot* 86:1474–1486.

Nyffeler R, Bayer C, Alverson WS (2005) Phylogenetic analysis of the Malvadendrina clade (*Malvaceae s.l.*) based on plastid DNA sequences. Org Divers Evol 5:109–123.

Tate JA, Fuertes J, Wagstaff SJ (2005) Phylogenetic relationships within the tribe Malveae (Malvaceae, subfamily Malvoideae) as inferred from ITS sequence data. *Am J Bot* 92:584-602.

MORACEAE

Datwyler SL, Weiblen GD (2004) On the origin of the fig: phylogenetic relationships of *Moraceae* from *ndhF* sequences. *Am J Bot* 91:767-777.

MUSACEAE

Christelová P, Valárik M, Hribová E et al. (2011) A multi gene sequence-based phylogeny of the *Musaceae* (banana) family. *BMC Evol Biol* 11:103.

MYRTACEAE

Biffin E, Lucas EJ, Craven LA et al. (2010) Evolution of exceptional species richness among lineages of fleshy-fruited *Myrtaceae*. *Ann Bot* 106:79–93.

NYCTAGINACEAE

Douglas NA, Manos PS (2007) Molecular phylogeny of *Nyctaginaceae*: taxonomy, biogeography, and characters associated with a radiation of xerophytic genera in North America. *Am J Bot* 94:856–872.

OLEACEAE

Wallander E, Albert VA (2000) Phylogeny and classification of *Oleaceae* based on *rps16* and *trnL-F* sequence data. *Am J Bot* 87:1827–1841.

PLANTAGINACEAE

Albach DC, Meudt HM, Oxelman B (2005) Piecing together the “new” *Plantaginaceae*. *Am J Bot* 92:297–315.

Rønsted N, Chase MW, Albach DC, Bello MA (2002) Phylogenetic relationships within *Plantago* (Plantaginaceae): evidence from nuclear ribosomal ITS and plastid trnL-F sequence data*. Bot J Linn Soc* 139:323–338.

POACEAE

Barker NP, Galley C, Verboom GA et al. (2007) The phylogeny of the austral grass subfamily Danthonioideae: evidence from multiple data sets. *Plant Syst Evol* 264:135–156.

Davis JI, Soreng RJ (2007) A preliminary phylogenetic analysis of the grass subfamily Pooideae (*Poaceae*), with attention to structural features of the plastid and nuclear genomes, including an intron loss in GBSSI. *Aliso* 23:335–348.

Giussani LM, Cota-Sánchez JH, Zuloaga FO, Kellogg EA (2001) A molecular phylogeny of the grass subfamily Panicoideae (*Poaceae*) shows multiple origins of C_4_ photosynthesis. *Am J Bot* 88:1993–2012.

Peterson PM, Romaschenko K, Johnson G (2010) A classification of Chloridoideae (*Poaceae*) based on multi-gene phylogenetic trees. *Mol Phylogenet Evol* 55:580–598.

Quintanar A, Castroviejo S, Catalán P (2007) Phylogeny of the tribe Aveneae (Pooideae, *Poaceae*) inferred from plastid *trmT-F* and nuclear ITS sequences. *Am J Bot* 94:1554–1569.

Soreng RJ, Peterson PM, Romaschenko K et al. (2015) A worldwide phylogenetic classification of the *Poaceae* (Gramineae). *J Syst Evol* 53:117–137.

Strauss SY, Webb CO, Salamin N (2006) Exotic taxa less related to native species are more invasive. *P Natl Acad Sci USA* 103:5841-5845.

POLYGONACEAE

Sanchez A (2011) Evolutionary relationships in *Polygonaceae* with emphasis in Triplaris. *PhD thesis*, Wake Forest University, North Carolina, USA.

PRIMULACEAE

Martins L, Oberprieler C, Hellwig FH (2003) A phylogenetic analysis of *Primulaceae* *s.l.* based on internal transcriber spacer (ITS) DNA sequence data. *Plant Syst Evol* 237:75–85.

ROSACEAE

Potter D, Eriksson T, Evans RC et al. (2007) Phylogeny and classification of *Rosaceae*. *Plant Syst Evol* 266:5–43.

RUBIACEAE

Bremer B, Eriksson T (2009) Time tree of *Rubiaceae*: Phylogeny and dating the family, subfamilies, and tribes. *Int J Plant Sci* 170:766–793.

SAPINDACEAE

Harrington M (2008) Phylogeny and evolutionary history of *Sapindaceae* and Dodonea. *PhD thesis*, James Cook University, Queensland, Australia.

SCROPHULARIACEAE

Olmstead RG, de Pamphilis CW, Wolfe AD et al. (2001) Disintegration of *Scrophulariaceae*. *Am J Bot* 88:348–361.

Oxelman B, Kornhall P, Olmstead RG, Bremer B (2005) Further disintegration of *Scrophulariaceae*. *Taxon* 54:411–425.

SOLANACEAE

Olmstead RG, Bohs L, Migid HA et al. (2008) A molecular phylogeny of the *Solanaceae*. *Taxon* 57:1159–1181.

URTICACEAE

Wu Z-Y, Monro AK, Milne RI et al. (2013) Molecular phylogeny of the nettle family (*Urticaceae*) inferred from multiple loci of three genomes and extensive generic sampling. *Mol Phyl Evol* 69:814–827.

VERBENACEAE

Marx HE, O’Leary N, Yuan Y-W et al. (2010) A molecular phylogeny and classification of *Verbenaceae*. *Am J Bot* 97:1647–1663.
