# Supplementary figures and images for: Invasions but not extinctions change phylogenetic diversity of angiosperm assemblage on southeastern Pacific Oceanic islands
Source: PLoS One. 2017 Aug 1;12(8):e0182105. doi: 10.1371/journal.pone.0182105 (PMC5538740; doi:10.1371/journal.pone.0182105)

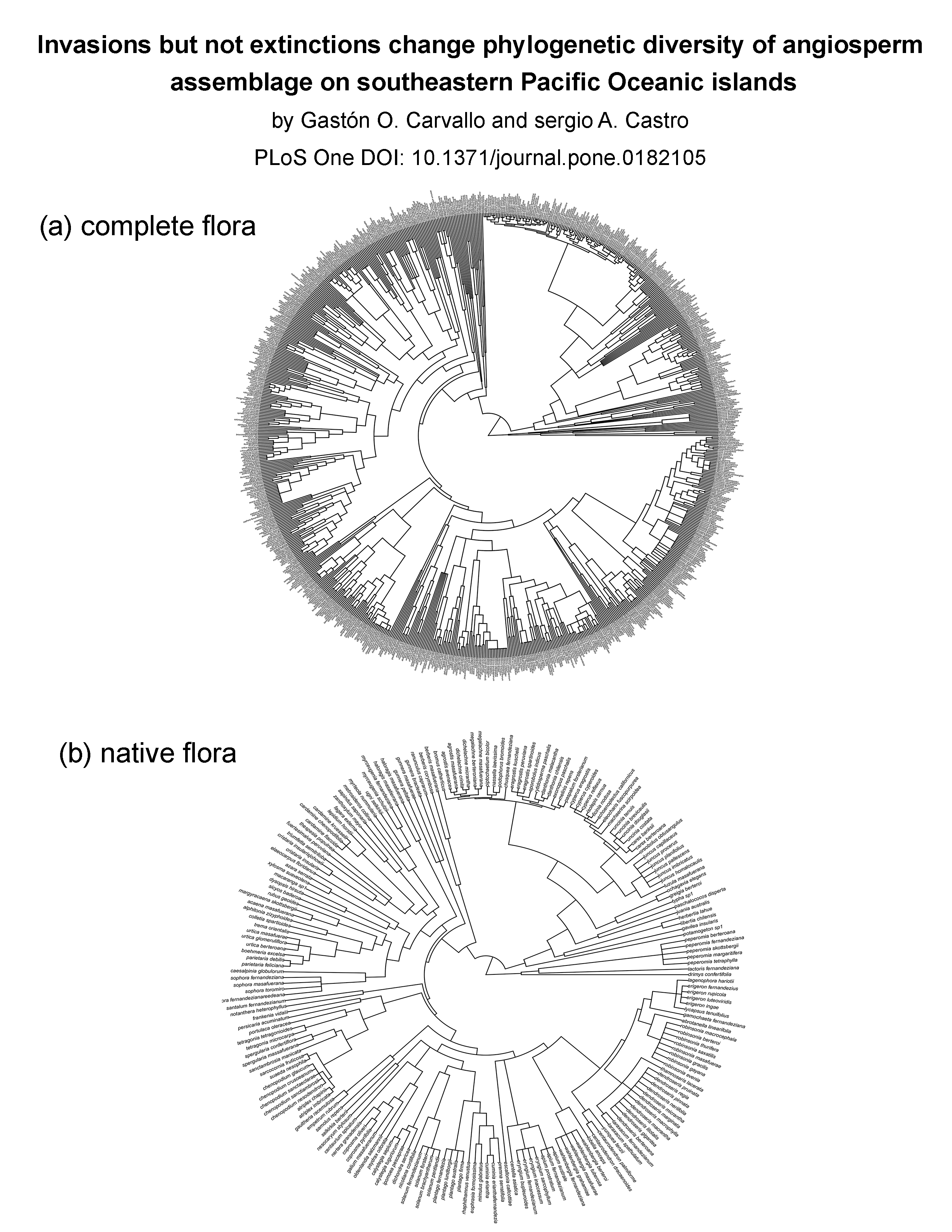

Supplement: S1 Fig — (TIF) [file pone.0182105.s003.tif]
